# Analgesic Effect of Dual Injection Technique for the Erector Spinae Plane Block in Beating Heart Coronary By-Pass Surgeries

**DOI:** 10.7759/cureus.14122

**Published:** 2021-03-26

**Authors:** Sami Kaan Coşarcan, Alper Tunga Doğan, Yavuz Gurkan, Ömür Erçelen

**Affiliations:** 1 Anesthesiology, Vehbi Koç Vakfı American Hospital, Istanbul, TUR; 2 Anesthesiology, Koç University, İstanbul, TUR

**Keywords:** regional anesthesia, coronary artery bypass surgery, postoperative pain, erector spinae plane block, mechanism of action erector spinae plane block

## Abstract

Introduction

Various regional anesthesia techniques such as thoracic epidural, thoracic paravertebral block, erector spinae plane block (ESPB), parasternal intercostal blocks are used in cardiac surgery for postoperative analgesia. In our study, we investigated the analgesic efficacy of the dual injection technique of ESPB in beating heart coronary bypass surgeries.

Methods

The records of patients with coronary artery bypass (CABG) surgery in the beating heart at the VKV American Hospital between January and December 2019 were retrospectively analyzed. The data of 30 patients who met the criteria to be included in the study were analyzed. Whether any opioid use is required for maintenance of anesthesia it is recorded. The pain scores of the patients are recorded by the intensive care team and cardiovascular service nurses for the first 48 hours.

Results

The absence of secondary responses to pain in all surgical periods, including skin incision and sternotomy, and low number of rating scale (NRS) scores in the postoperative 0- to 24-hour period show that the technique we developed can produce effective analgesia. After the 24th postoperative hour, the patients were followed up in the cardiovascular service and there was no opioid use between 24- to 48-hour period.

Conclusion

Our approach, in which the local anesthetic is applied by approaching the superior costa-transverse ligament (SCTL) in the ESPB, provides an effective analgesia in coronary artery bypass surgeries in the beating heart. The main purpose of our new approach is to increase the amount of local anesthetic in the paravertebral area. We recommend using our modified technique for effective analgesia after CABG surgeries.

## Introduction

Cardiac surgery causes moderate and/or severe pain due to sternotomy, sternal retraction, internal mammarian artery removal and chest tube placement. Failure to manage pain sufficiently causes systemic and pulmonary complications. Tachycardia and hypertension attacks that develop in response to pain increase myocardial oxygen consumption and cause hemodynamic disturbances [1].

Effective analgesia is important in creating optimal surgical conditions, especially in coronary artery bypass surgeries (CABG) performed on the beating heart. Enhanced recovery after surgery (ERAS) protocols aim to reduce opioid use. This goal can be reached more easily by using regional analgesia methods. Severe chronic pain after sternotomy is shown as approximately 35% in the first year [2]. With the increased awareness of opioid dependence, excessive or abusive use of opioids, and tolerance-hyperalgesia, limited use of opioids or opioid-sparing modalities have been developed [3]. The most important step in opioid-sparing analgesia management is the use of regional anesthesia techniques. Various regional anesthesia techniques such as thoracic epidural, thoracic paravertebral block, parasternal intercostal blocks are used in cardiac surgery for postoperative analgesia. Coagulation problems make it difficult and restrict the implementation of central blocks. Ultrasound-guided fascial plan blocks are also gaining popularity in cardiac surgeries [4]. For this purpose, erector spinae plan block (ESPB) is widely used, but different and inconsistent clinical results are observed. Recently we have introduced our dual injection ESPB technique which today we use routinely in our clinic.

In our clinic, CABG surgeries are performed mostly in the beating heart, and providing effective analgesia in the intraoperative period enables surgery to be performed under optimal conditions. We frequently use regional anesthesia techniques in terms of effective analgesia management. In our study, we investigated the effectiveness of ESP block modification in terms of both perioperative and postoperative analgesia with the dual injection technique we described for CABG surgery in the beating heart.

## Materials and methods

After the approval of Koç University Clinical Research Ethics Committee (2020,019.IRB1.010), the records of patients with CABG surgery in the beating heart at the VKV American Hospital between January and December 2019 were retrospectively analyzed. Patients with a history of cerebral events, a history of Alzheimer's-dementia, planned for carotid surgery, and undergoing CABG surgery under emergency or semi-emergency conditions were excluded from the study. Patients who were found to have applied any regional anesthesia method other than ESPB in the examined files were not included in the study.

Perioperative anesthesia management

In open-heart surgery operations, Bispectral Index (BIS, Vista) and INVOS (Medtronic, Minneapolis, USA) parameters are routinely used in our clinic. General anesthesia induction and maintenance of the patients are performed under the same protocol with the BIS and INVOS parameters.

In our clinic, general anesthesia was performed with 2 mg·kg^−1^ propofol, 1 μg·kg^−1^ fentanyl, 0.6 mg·kg^−1^ rocuronium. Anesthesia was maintained by remifentanil infusion 0-250 µg/kg/min and 0.5-1 MAC sevoflurane. We limited the amount of opioids in anesthesia management and postoperative pain management. Anesthesia procedures are initiated in the operation room after all patients have received their informed consent at least 24 hours before. Whether any opioid use is required for maintenance of anesthesia it is recorded. Unless there are contraindications, all patients are extubated in the operation room and transferred to the intensive care unit. The pain scores of the patients are recorded by the intensive care team and cardiovascular service nurses for the first 48 hours.

Dual injection technique for the erector spinae plane block (DIESPB)

**Figure 1:**
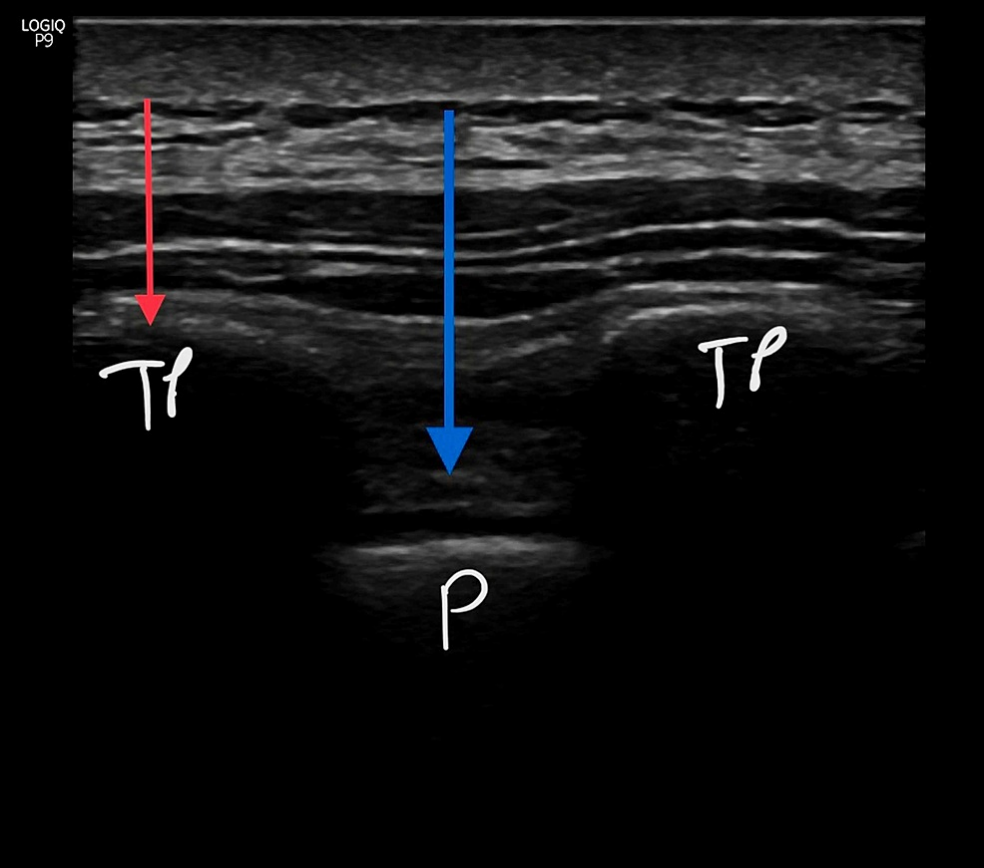
Ultrasound-guided dual injection technique for the erector spinae plane block. SCTL: superior costa-transverse ligament; ESPB: erector spinae plane block; TP: transverse process; P: pleura; red arrow: first injection point – classical ESPB; blue arrow: second injection point – above the SCTL.

**Figure 2:**
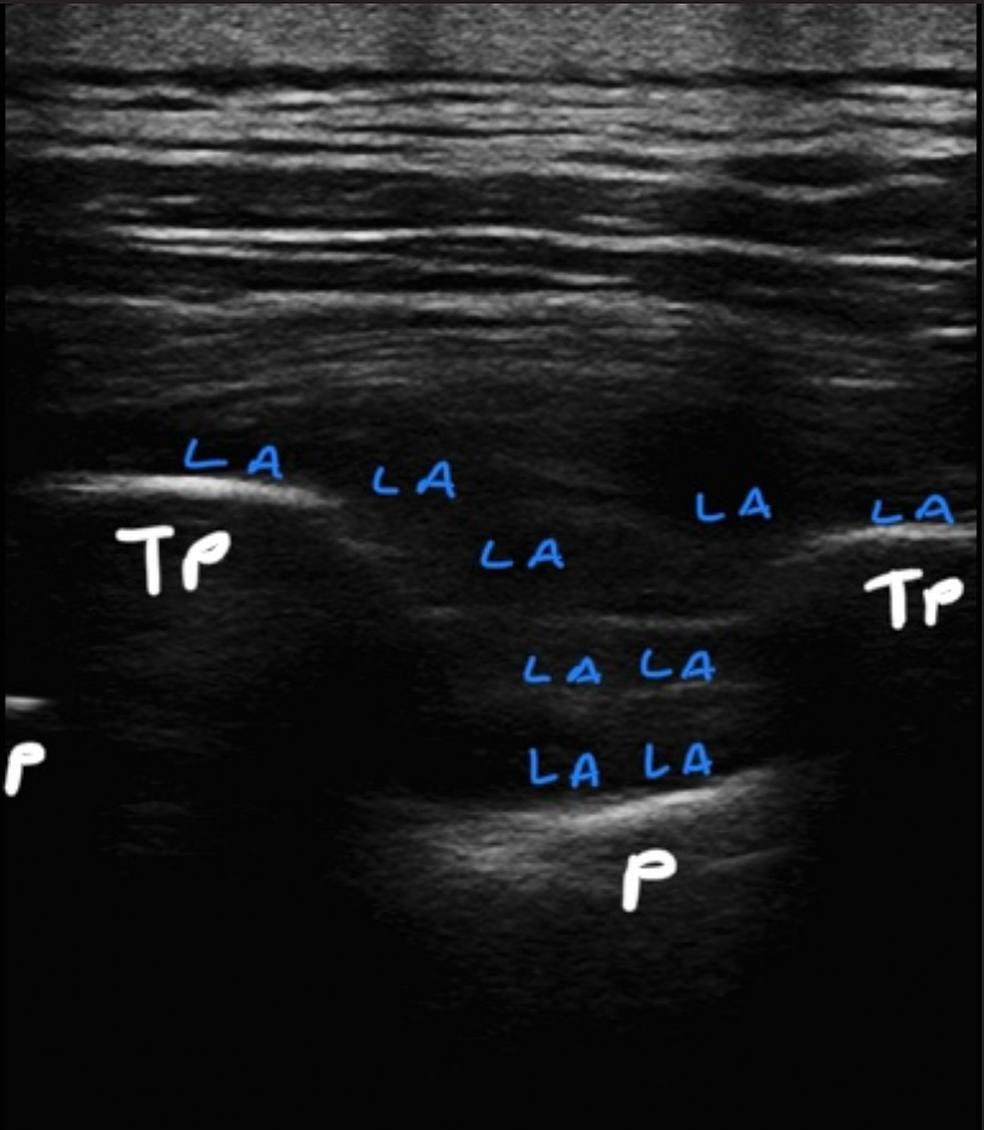
Push-down view of the pleura and local anesthetic distribution (above the TP and inside the intertransverse connective tissue complex) under ultrasound. TP: transverse process; P: pleura; LA: local anesthetic.

Anesthesia procedures are initiated in the operating room after all patients have received their informed consent at least 24 hours before the preoperative period. Routinely we use ESPB's dual injection technique before induction of the general anesthesia. In a sitting position, followed by sterile staining ultrasound with a linear probe (GE Healthcare (General Electric Company), USA Logiq P9, L4-12t-RS 4.2-13 mHz) placed next to thoracic 3-4 (T3-4). Transverse process of T4 is determined. 80 mm needle (BBraun Melsungen, Germany, Ultra360 stimuplex) is advanced in-plane in the cauda-cranial direction. After the needle contact to the transverse process, hydrodissection with serum saline between the erector spina muscle and fasciae is confirmed, 10 mL of 0.350% bupivacaine is given. Subsequently, the needle is advanced through both transverse processes and the intertransverse ligament. After fascial spreading is observed with saline hydrodissection method the remaining 15 mL of 0.350 bupivacaine is given above the superior costa-transverse ligament (SCTL). The same procedure is applied to the other side (Figures 1, 2).

## Results

**Figure 3:**
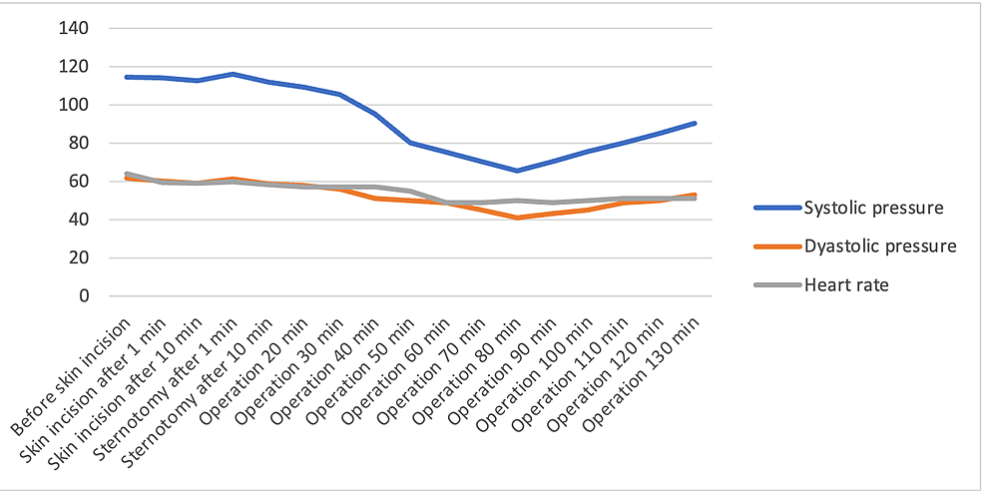
Hemodynamic response (skin incision and sternotomy) (mean values).

**Figure 4:**
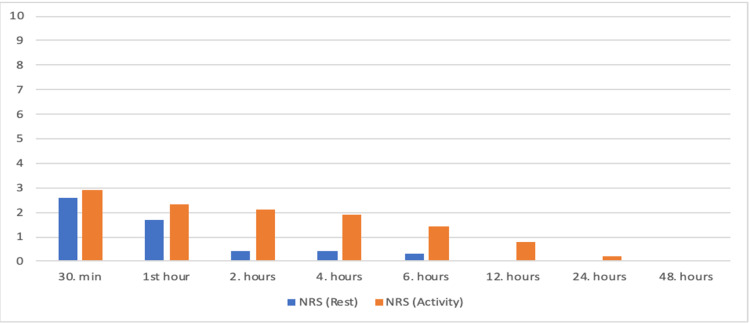
NRS values (at rest/during activity) (mean values). NRS: number of rating scale.

The files of 60 patients who underwent CABG surgery in the beating heart in our clinic between January and December 2019 were retrospectively analyzed. Of these, thirteen patients who had critical main coronary artery lesions were excluded from the study. Ten patients with a history of cerebral events and planned to have carotid surgery were excluded. Seven patients who were found to have incomplete records of pain at intensive care/cardiovascular service were also excluded from the study. The data of 30 patients who met the criteria to be included in the study were analyzed. The demographic data and operation features of the patients are shown in Table 1. It was observed that there was no response due to surgical stimulus at 0, 1 and 10 minutes after skin incision and 1 and 10 minutes after sternotomy (Figure 3). In the files examined, it was observed that no additional opioid analgesia was applied to the patients during the intraoperative period. It was determined that 1,000 milligram (mg) paracetamol (PAROL 100 ml, 10mg/ml, ATABAY) and 50 milligram (mg) tramadol (CONTRAMAL, ABDI IBRAHIM) were administered to all patients before extubation. Pain scores during 30 min, 1-hour, 2-hour, 4-hour, 6-hour, 12-hour, 18-hour, 24-hour and 48-hour number of rating scale (NRS) rest and activity after extubation are shown in Figure 4. (Low NRS scores in the postoperative 48-hour period.) During the follow-up in the coronary intensive care unit and service, 3x1 1000 milligram paracetamol was used as a routine analgesic, and the additional analgesic type and usage rates other than paracetamol are shown in Table 2 (very low additional opioid use in the postoperative period). It was observed that no postoperative patient was admitted to non-invasive or invasive mechanical ventilator support. It was determined that the patients were mobilized and transferred to the cardiovascular surgery service without any problems at the 24th hour postoperatively.

**Table 1:** Demographic data and operation features (number of patients/mean values‪ ± SD)‬‬‬‬‬‬‬‬‬‬‬‬‬‬‬‬‬‬.

Gender (M/F)	20/10
Age (years)	56 ‪± 19.4
Height (cm)	165.7‪ ‪‪± 5.3
Weight (kg)	76.8 ‪± 9.8
Diabetes mellitus (DM) (+/-)	20/10
Hypertension (HT) (+/-)	27/3
Operation time (min)	135.7 ‪± 36.2
Preoperative morphine use (mg)	2.2 ‪±1.5
Right internal mammary artery (RIMA) (+/-)	19/11
Left internal mammary artery (LIMA) (+/-)	30/0
Radial artery (+/-)	21/9

**Table 2:** Postoperative total opioid consumption doses (mean values ‪± SD).‬‬‬‬‬‬‬‬‬‬‬‬‬‬‬‬ (Very low additional opioid use in the postoperative period) (mg:milligram)

Total opioid amount (mg)	Tramadol	Morphine	Meperidine
Postoperative 24-hour	51.38 ± 18.13	0	32.77 ± 11.14
Postoperative 24-48 hour	0	0	0

## Discussion

In the one-year cohort study published by Lahtinen et al. [5], it was shown that 48% of patients felt severe pain at rest, 62% experienced severe pain with movement, and 78% experienced severe pain with cough after movement during the postoperative four-day period after cardiac surgery. Chronic pain that can be seen after sternotomy also adversely affects post-hospital comfort. The incidence of chronic pain is more common, especially in patients with LIMA removal. If acute pain cannot be managed optimally, chronic pain is observed more frequently. Therefore, the ideal management of acute pain is very important in terms of patient comfort and morbidity in the short postoperative period and prevention of chronic pain in the long term [6-9].

An inconsistent relationship between cerebral oxygenation and sympathetic block has been demonstrated. It is stated that the sympathetic block occurred with the cervical plexus block, the cerebral blood flow increases on the same side but there may be a decrease in the cerebral blood flow on the opposite side [6]. In addition, increased cerebral blood flow may be beneficial in the management of the hypotensive anesthesia required for CABG operations especially performed in the beating heart.

Various fascial plane blocks are used in open-heart surgeries performed with sternotomy. The pectoral block (PECS I and PECS II) provides analgesia in the lateral thoracic wall through the lateral pectoral nerves [4,10]. Although the serratus plan block provides a larger area of ​​analgesia than PECS blocks, these blocks are ineffective in sternotomy pain in open-heart surgery. The nerves that cause sternotomy pain are a complex innervation network that includes the T2-T6 dermatomes and is also accompanied by anterior cutaneous nerves of ventral ramus origin [4,10,11]. The ESPB is a novel plane block that was defined by Forrero in 2016, demonstrating analgesic activity in thoracic and abdominal surgery. The mechanism of action is thought to be as a result of spreading of local anesthetic between the erector spina muscle and the fascia of the transverse process reaching the intervertebral foramen, by affecting the dorsal and ventral branches of the spinal nerves [12]. Although the mechanism of action has not been fully revealed, the involvement of the dorsal ramies of the spinal nerves and the cauda-cranial spread is anticipated as the main mechanism. Although some studies have shown that ventral ramies are affected, most anatomical studies show that the cutaneous branches of the paravertebral area, epidural area and intercostal nerves do not have involvement. Failure to block the lower cervical dermatomes innervating the upper part of the sternum during ESPB results in partial deficiency of these blocks [4,13,14]. Anatomic studies have shown that there is no spreading to the paravertebral area in the ESPB and SCTL can play a key role at this point [15-17]. Therefore, different modifications are proposed to make the management of pain more optimal in median sternotomy incisions such as with cardiac surgery. As stated by Tulgar et al. [18], various techniques described near the transverse processes can be called as different techniques rather than being variants of ESPB. The variations developed are due to efforts to improve weak spots in terms of ESPB’s clinical efficacy. Costache et al. defined the mid-point transverse process to pleura (MTP) block and identified the midpoint between the intertransverse ligament and the pleura as the injection site [19]. Nielsen et al. identified the costatransverse block by specifying the injection site just before the contact of the lower rib after the intertransverse ligament passed [20]. In all ESPB variants described are aimed to increase the passage of local anesthetics to the paravertebral area. In our clinic, we defined the dual injection technique in ESPB. In addition to the classic ESPB, this novel approach suggests a second injection above SCTL under the intertransverse ligament [21].

There is an anatomical structure called the intertransverse connective tissue complex between the erector spinae plane and the paravertabral space. In this complex, there are intertransverse ligaments, costotransverse ligaments, levator costarum, rototor costarum, external intercostal muscles and adipose tissue. In this complex, it is thought that the local anesthetic has a structure that allows passage to the paravertebral area, and even the pressure of the local anesthetic can play a role in this transition [22]. In addition to the conventionally defined ESPB, it is shown to be more effective to add the injection made between the intertransverse connective tissue complex above the SCTL [23].

For thoracic wall analgesia, being close to the paravertebral area may be important for effectiveness. It is very important to be able to block the lateral cutaneous and anterior cutaneous nerves, which are the branches of the ventral ramus, especially when the median sternotomy, such as cardiac surgery, and where the chest wall anterior-middle innervation becomes important. From this point of view, classic ESPB, may not offer a very effective analgesia in cardiac surgery operations involving sternotomy. The importance of the role of ESPB in multimodal analgesia is mentioned in articles in this field [24-26]. Although Tsui et al. [27] showed the effect of ESPB in a CABG operation, they reported the use of high-dose fentanyl during the operation and the use of additional analgesics in the postoperative period shows it is effective in multimodal analgesia, but its effect cannot be significant alone. Regardless of the technique used in cardiac surgery, adding the regional anesthesia method has been shown to give better results than traditional opioid use. Although studies focus on ESPB, it is a fact that we need blocks with longer duration of action and more randomized studies [28]. After open heart operations, approximately 3.3% of patients may continue using opioid even after 90 days. Hyperalgesia due to opioid use can be an important problem and remifentanil/fentanyl infusions used during the operation may also cause this situation [29,30]. ESPB studies in coronary artery bypass surgeries are few and mostly in the form of case reports. In the presented cases, it emphasizes the effectiveness of the postoperative period and its contribution to early extubation rather than the perioperative analgesic efficacy. In our study, the effectiveness of the intraoperative period was tried to be shown with hemodynamic parameters. Its contribution to hemodynamic stability during operation is shown without the use of additional opioids. The absence of secondary responses to pain in all surgical periods, including skin incision and sternotomy, and low NRS scores in the postoperative 48-hour period show that the technique we developed can produce effective analgesia. The patients did not take any opioids during their time in the cardiovascular service. It was observed, that patients used only paracetamol during this period. Even the NRS activity (during coughing and/or breathing exercise) scores at a level that does not require additional analgesic intake in the postoperative period shows that highly effective results are obtained compared to other CABG studies. As shown in our study, we think that the reduced usage of additional opioids in the postoperative period can contribute significantly to early recovery and rehabilitation in CABG.

Our approach, in which the local anesthetic is applied by approaching the superior costa-transverse ligament (SCTL) in the erector spina plane block, provides an effective analgesia in coronary artery bypass surgeries in the beating heart. In the light of the studies conducted in the classical-defined form of the erector spinae plane block, our dual injection technique, which is closer to SCTL combined with the ESPB, in order to increase the anterior spread, create an effective and long-term block and increase the amount of local anesthetic into the paravertebral area. 

The main purpose of our new approach is to increase the amount of local anesthetic in the paravertebral area. We think that keeping the needle on the SCTL may be safer in terms of complications. We think that the spread of local anesthetic within the intertransverse tissue complex will play a key role in the transition to the paravertebral area. According to our clinical experience, dual injection technique seems to be effective in cardiac surgery. We recommend using our dual injection technique for effective analgesia after CABG surgeries. 

Limitations

This study is a retrospective study. Any regional anesthesia technique to compare with the modified ESPB was not used. Comparison between the classically defined ESPB and the modified ESPB effects we use has not been made. In our clinic, regional anesthesia techniques are used in all elective bypass surgeries. Therefore, no comparison was made with patients without regional anesthesia technique. There is no group to compare in the study so the statistical analysis was not performed.

## Conclusions

In our study, it was observed that the dual injection technique for the ESPB was clinically effective in open-heart surgery. This novel approach needs to be tested in large patient series.
